# Inter- and intrafraction dose variations in robotic stereotactic body radiation therapy (SBRT) for perihilar cholangiocarcinoma in the prospective phase I STRONG trial

**DOI:** 10.3389/fonc.2023.1114737

**Published:** 2023-03-08

**Authors:** Chiara Paronetto, Wilhelm den Toom, Maaike T. W. Milder, Yvette van Norden, Rogier Baak, Ben J. M. Heijmen, Alejandra Méndez Romero

**Affiliations:** ^1^ Department of Radiotherapy, Erasmus MC Cancer Institute, University Medical Center Rotterdam, Rotterdam, Netherlands; ^2^ Department of Radiotherapy, Istituto Oncologico Veneto (IOV), Padova, Italy

**Keywords:** perihilar cholangiocarcinoma, robotic tumor tracking, stereotactic body radiation therapy (SBRT), Inter- and intrafraction dose variations, adaptive radiotherapy (ART)

## Abstract

Using fiducial-marker-based robotic respiratory tumor tracking, we treated perihilar cholangiocarcinoma patients in the STRONG trial with 15 daily fractions of 4 Gy. For each of the included patients, in-room diagnostic-quality repeat CTs (rCT) were acquired pre- and post-dose delivery in 6 treatment fractions to analyze inter- and intrafraction dose variations. Planning CTs (pCTs) and rCTs were acquired in expiration breath-hold. Analogous to treatment, spine and fiducials were used to register rCTs with pCTs. In each rCT, all OARs were contoured, and the target was rigidly copied from the pCT based on grey values. The rCTs acquired were used to calculate the doses to be delivered through the treatment-unit settings. On average, target doses in rCTs and pCTs were similar. However, due to target displacements relative to the fiducials in rCTs, 10% of the rCTs showed PTV coverage losses of >10%. Although target coverages had been planned below desired values in order to protect OARs, many pre-rCTs contained OAR constraint violations: 44.4% for the 6 major constraints. Most OAR dose differences between pre- and post-rCTs were not statistically significant. The dose deviations observed in repeat CTs represent opportunities for more advanced adaptive approaches to enhancing SBRT treatment quality.

## 1 Introduction

Perihilar cholangocarcinoma (pCCa) is the most frequent type of cholangiocarcinoma (CCa), and accounts for 50-70% of all cases (1). The standard treatment for unresectable pCCa is chemotherapy (2–4).

The recent addition of PDL1 inhibitors has shown good results for both OS and PFS (5, 6). Promising local control rates have also been shown by the few studies that investigated stereotactic body radiation therapy (SBRT) for cholangiocarcinoma (7–14). A recent retrospective study also proved that SBRT has good local control and overall survival without increased toxicity (15). Even though this data is not specific for pCCa, these results warrant further studies in this category of patients.

pCCa tumors are subject to respiratory motion and have extensions outside the liver that are close to many sensitive organs at risk (OARs). To the best of our knowledge, no published studies have investigated the dosimetric impact of inter- and intra-fraction anatomy variations for these tumors.

The STRONG study (NCT03307538) was the first prospective trial designed to evaluate the feasibility and toxicity of SBRT after first-line chemotherapy in patients with unresectable pCCa (16, 17). Its secondary endpoints were local control, progression-free survival, overall survival, and quality of life (QoL). No dose-limiting toxicity was observed; at a median follow-up of 14 months, 12-month local control was 80%; and QoL did not change (16). An important aspect of the trial was study of the dosimetric impact of inter- and intra-fraction anatomical variations through daily acquisition of repeat CTs (rCT) with an in-room CT on-rails, both pre- and post- daily dose delivery. In this paper we report deviations of delivered target and OAR doses in rCT anatomies from planned doses established with the planning CT (pCT). Furthermore, pre-rCT and post-rCT doses were mutually compared to assess intra-fraction dose variation in the investigated patient population.

## 2 Materials and methods

### 2.1 Patients and clinical workflow

The six patients (three male and three female) in the STRONG trial were included in this study. All had stage cT4 disease, with three cN0 and the other three cN1. The median tumor size was 26 mm, which ranged from 21 to 36 mm. Five of the six patients had a stent in the biliary tree that passed through the GTV.

Patients were treated with SBRT using Synchrony^®^ respiratory tracking with the robotic M6 CyberKnife^®^ (Accuray Inc., Sunnyvale, CA, USA), which was equipped with an Incise MLC. To this purpose, two fiducial markers (Tornado^®^ Embolization Coils, Bloomington, USA) were implanted in the liver of each patient, close to the intrahepatic component of the tumor. Both during acquisition of pCTs and rCTs) and during dose delivery, patients were immobilized in a vacuum mattress. The total dose was delivered in 15 fractions of 4 Gy prescribed at the 80% isodose line.

To establish planning contours, we acquired not only a 4DCT scan but, after patient instruction, also voluntary expiration and inspiration CT scans with IV contrast. In the expiration CT we contoured all OARs (healthy liver, heart, spinal cord, kidneys, gallbladder, central biliary tract, stomach, esophagus, duodenum, bowel, liver). After contouring the first patient, OARs close to the target were also contoured in the inspiration CT. To avoid constraint violations in the two scans, account was taken of these contours during planning. Contouring of the GTV = CTV on the expiration CT was supported by additional information acquired by a gadolinium-enhanced liver MRI, and the contour was reviewed with an expert radiologist. For all patients we used a PTV margin of 7 mm, which was established using 4DCT scans, taking account of the distances observed between the fiducials’ center-of-mass and the target center (18).

The planning aim was to cover 95% of the PTV with the prescribed 4Gy per fraction. However, during planning, priority was given to avoiding violations of the OAR constraints, which might reduce PTV coverage. The OAR planning constraints applied are described in detail in the study protocol and in **Table 1** (16, 17). During treatment, the same planning CT (pCT) was used to make a new plan for two of the six patients.

**Table 1:** 1st data column: Mean dosimetric GTV, PTV and OAR plan parameter values in the pCT for the 6 study patients. 2nd data column: Plan parameter differences between pre-rCTs and corresponding pCTs.

Structure parameter + constraint^*^	pCT parameter value mean ± SD, range	pre-rCT – pCT mean ± SD, range P-value	pre-rCT – post-rCT mean ± SD, range P-value
GTV V60Gy (%)	91.1 ± 5.8, [82.2,98.6]	2.5 ± 3.3 [-8.2,8.1] **<0.0001**	2.2 ± 3.0 [-2.1,9.8] **0.0002**
GTV V45Gy (%)	98.3 ± 2.5, [93.6,100]	0.6 ± 1.0 [-0.37,4.3] **0.002**	0.7 ± 1.4 [-0.7,5.1] **0.006**
GTV D98% (Gy)	50.6 ± 7.9, [40.0,61.8]	2.8 ± 3.8 [-11,8.7] **0.0001**	2.0 ± 2.9, [-3.3,14.0] **0.0004**
GTV D2% (Gy)	72.7 ± 1.4, [70.4,74.2]	2.0 ± 1.6, [-0.9,7.1] **<0.0001**	0.37 ± 1.7, [-1.1,9.7] 0.2
PTV V60Gy (%)	83.1 ± 5.2, [75.8, 90.0]	-0.8 ± 4.4 [-18.0,4.2] 0.3	2.1 ± 3.9, [-3.5,12.9] **0.003**
PTV V45Gy (%)	95.0 ± 3.6 [89.0,99.3]	0.77 ± 2.1 [-5.9, 4.1] **0.04**	1.9 ± 3.3 [-1.7,13.4] **0.002**
PTV D98% (Gy)	42.3 ± 3.7, [38.5,49.1]	1.0 ± 4.4, [-14,13.4] 0.2	3.1 ± 6.1 [-2.8, 23.7] **0.005**
PTV D2% (Gy)	72.4 ± 1.2, [70.8,73.6]	1.85 ± 1.6, [-0.97,7.84] **<0.0001**	0.07 ± 0.57, [-1.3,1.1] 0.4
Stomach V41Gy ≤ 5 cc	1.6 ± 1.6, [0.0,3.8]	2.0 ± 3.1, [-1.5,8.8] **0.0005**	0.64 ± 1.7, [-3.6,4.7] **0.03**
Stomach Dmax < 57 Gy	46.1 ± 9.8 [29.3,57.3]	3.9 ± 10.4 [-30,21.9] **0.03**	0.3 ± 6.4 [-28,12.3] 0.8
Duodenum V41Gy ≤ 5 cc	2.8 ± 1.3 [1.0,4.5]	1.3 ± 3.8 [-2.5,10.8] **0.05**	0.5 ± 2.1 [-4.2,5.7] 0.1
Duodenum Dmax < 57 Gy	53.0 ± 4.4 [47.0,57.9]	4.5 ± 9.3 [-7.5,21.5] **0.008**	-0.14 ± 5.8 [-9.4,13.1] 0.9
Gallbladder Dmax ≤ 63 Gy	59.3 ± 6.1 [48.9,63.6]	2.6 ± 8.3, [-34,14.4] 0.1	1.2 ± 10.5, [-34,26.3] 0.5
Central biliary tract D0.5cc < 70 Gy	68.2 ± 1.9 [64.4,69.7]	0.95 ± 2.7 [-7.4,8.2] **0.05**	-0.2 ± 1.3, [-3.1,3.0] 0.3

*Constraints were used only for OARs.

3rd data column: plan parameter differences between daily pre-rCTs and post-rCTs. P-values <0.05 are indicated in bold. The planning aim for PTV V60Gy was 95%.

Before each treatment fraction, skin marks were used to position the patient on the robotic treatment couch. Next, the patient was further aligned using two orthogonal kV images to perform a spine match with the pCT, consisting of translations and rotations. Finally, patient setup was further adjusted on the basis of a fiducial match consisting of only translations. During tracking, correspondence models were updated every 150 sec.

On treatment days, patients were asked not to consume solid food two hours before treatment. To maximize anatomical consistency, radiotherapy was delivered whenever possible in the time slot that was also used to acquire the pCT.

### 2.2 Acquisition of rCTs

Per patient, we acquired a pre-rCT and post-rCT in 6 of the 15 treatment fractions. For this we used an in-room on-rail SOMATOM Definition AS CT scanner (Siemens Healthcare, Forchheim, Germany) that was integrated into the stereotactic treatment unit together with a common robotic treatment couch (19). Scans were acquired in treatment position in expiration without IV contrast. Information collected from the rCTs was not used for actual treatments.

### 2.3 Target and OAR contours in rCTs

For our analyses, pCTs with the clinical contours, and acquired rCTs were imported in MIM version 6.9 (MIM Software Inc Cleveland OH). Due to the lack of IV contrast in rCTs, we considered manual contouring of GTVs in rCTs to be unfeasible. We therefore mapped pCT-GTVs on rCTs, based on automatic rigid rCT-pCT translation-rotation registrations on grey values in the PTV. After a pCT-GTV contour had been copied into an rCT, a 7 mm PTV margin was added, as in clinical planning. The validity of the copied GTVs was verified by checking the position of the stents and the surrounding tissues.

OARs were manually contoured in rCTs using the contours in the corresponding pCT as visual reference. Although, in principle, a stent positioned in the biliary tract was contoured as part of the biliary tract, we considered the stent and cystic duct in each of the 5 patients who had stents to be integral to the central biliary tract. All OARs were first contoured by a radiation oncologist (CP) and later reviewed and possibly adjusted in discussions with a second expert radiation oncologist (AMR).

### 2.4 rCT dose calculations

Before doses were calculated in the rCTs, the rCTs were registered to their respective pCTs in a two-step procedure similar to the daily tumor setup procedure (see above), in which a spine match (translations and rotations) was followed by a match of fiducials (translations only). To determine which dose had actually been delivered during treatment, we used a standalone version of the clinical dose engine to calculate the rCT dose distributions for the planned Cyberknife settings (such as MLC segment shapes, MUs, and beam angles) (20, 21). The clinical and standalone dose engines had slight differences in beam models. To establish a correction factor for the doses calculated with the standalone engine, the clinical planning CT dose distributions were first recalculated with the standalone engine, and the applied correction factor was then based on the requirement of equal PTV coverage at 60 Gy for clinical and standalone dose distributions.

### 2.5 Evaluation of rCT doses

The rCT dose distributions were evaluated for dosimetric parameters that were also used for treatment planning, with highlights on differences between pCTs and pre-rCTs, and between pre-rCTs and post-rCTs, and on violations of OAR planning constraints. If inhale and exhale OAR contours had both been used for planning (see M&M), rCT OAR dose parameters were compared only with the corresponding pCT values pertaining to the exhale scan.

### 2.6 Statistical analyses

The data were summarized using descriptive statistics. A T test was used to test the differences between pre-CT and plan CT, and pre- and post-treatment measures.

## 3 Results

**Supplementary Table S1** summarizes all treatment plan parameter values for all patients, including GTV, PTV and OARs for the pCT, pre-rCTs, and post-rCTs. As the doses in the heart, kidneys, spinal cord, bowel, esophagus and healthy liver were consistently far below constraint levels, the OAR analyses in the remainder of this paper focus on the central biliary tract, gallbladder, stomach and duodenum.

According to the STRONG study protocol, rCTs should have been acquired on days 1, 3, 6, 9, 12 and 15, but due to scheduling issues this was not always possible. The planned number of rCTs was nonetheless achieved for all patients: 6 pre-rCTs and 6 post-rCTs per patient. Due to incorrect couch height, two of the 72 rCTs had been wrongly acquired and were therefore discarded (patient 1, fraction 3, pre- and post-treatment), leaving 70 rCTs and 6 pCTs for analysis.

The correction factor needed to correct rCT doses to account for small differences in beam models between the clinical and the standalone dose calculation engine was 2.0%. Before the dosimetric analyses, all calculated rCT doses were therefore scaled with this value.

### 3.1 Target-dose deviations

**Table 1** (1st data column) and **Figure 1** show that the PTV coverage aims could not always be met during planning. On average, PTV V60Gy was 83.1 ± 5.2% in pCTs, with minimum and maximum values of 75.8% and 90% respectively, instead of the aimed 95%. Neither were the GTVs always fully covered with 60 Gy in pCTs: the mean GTV V60Gy was 91.1%, with an inter-patient range of [82.2,98.6]. Average PTV and GTV V45Gy in pCTs were 95.0% and 98.3%, respectively. Here, too, the respective ranges [89.0,99.3] and [93.6,100] show that inter-patient spreads were substantial (**Table 1**).

**Figure 1:**
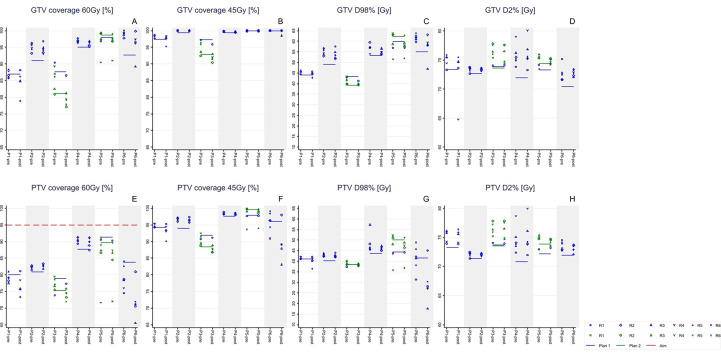
For all 6 study patients (P_i_, i=1..6), re-calculated GTV (upper panels **A-D**) and PTV (lower panels **E-H**) doses in pre- and post-rCTs (markers) compared to planning (horizontal lines). During the fractionated treatment, the initial treatment plan for patients 3 and 5 (plan 1, blue) was updated (plan 2, green). Rj (j=1,...6): rCT in the j-th fraction in which rCTs were acquired. Each panel describes an aim for the GTV and PTV as per STRONG study (see **Table 1**).

On average, deviations from planning of pre-rCT PTV (**Table 1**, 2nd data column) and GTV plan parameters (data rows 1-8, and **Figure 1**) were small (and, in percentage terms, much smaller than for OARs; see below). For example, analysis of all acquired 35 pre-rCTs together showed that, on average, PTV V60Gy was reduced by 0.8± 4.4% relative to planning (**Table 1**). **Figure 1** shows the inter-patient and day-to-day variations between pre-treatment scanning and planning for eight target-plan parameters. Comparisons of the last two data columns in **Table 1** and **Figure 1** show that the magnitude of differences between pre-rCT and post-rCT target plan parameters were similar to the magnitudes of difference between pre-rCT and pCT parameters.

**Figure 2:**
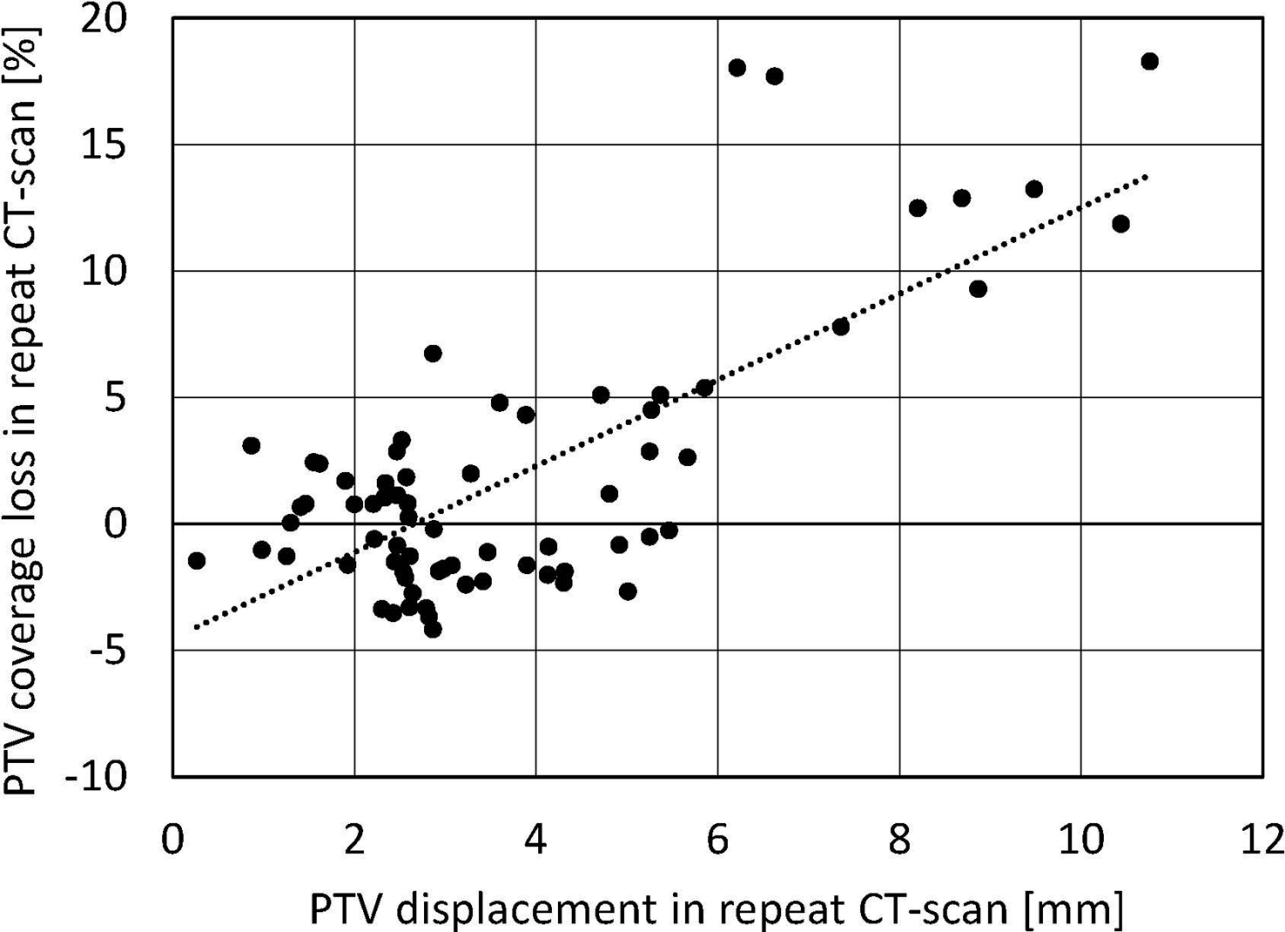
PTV coverage loss in repeat CT scans as a function of PTV displacement relative to fiducials. Each data point refers to one of the repeat scans of one of the included patients (70 data points in total). The dotted line is a linear fit.

**Figure 2** presents deviations in rCT PTV V60Gy from planning as a function of target displacement in a rCT relative to the center-of-mass of the corresponding fiducial. It shows clearly that greater target displacements led to greater coverage losses. Target displacements were likely caused by non-rigid anatomy changes between pCTs and rCTs, although the possibility of some marker migration cannot be excluded.

**Figure 3:**
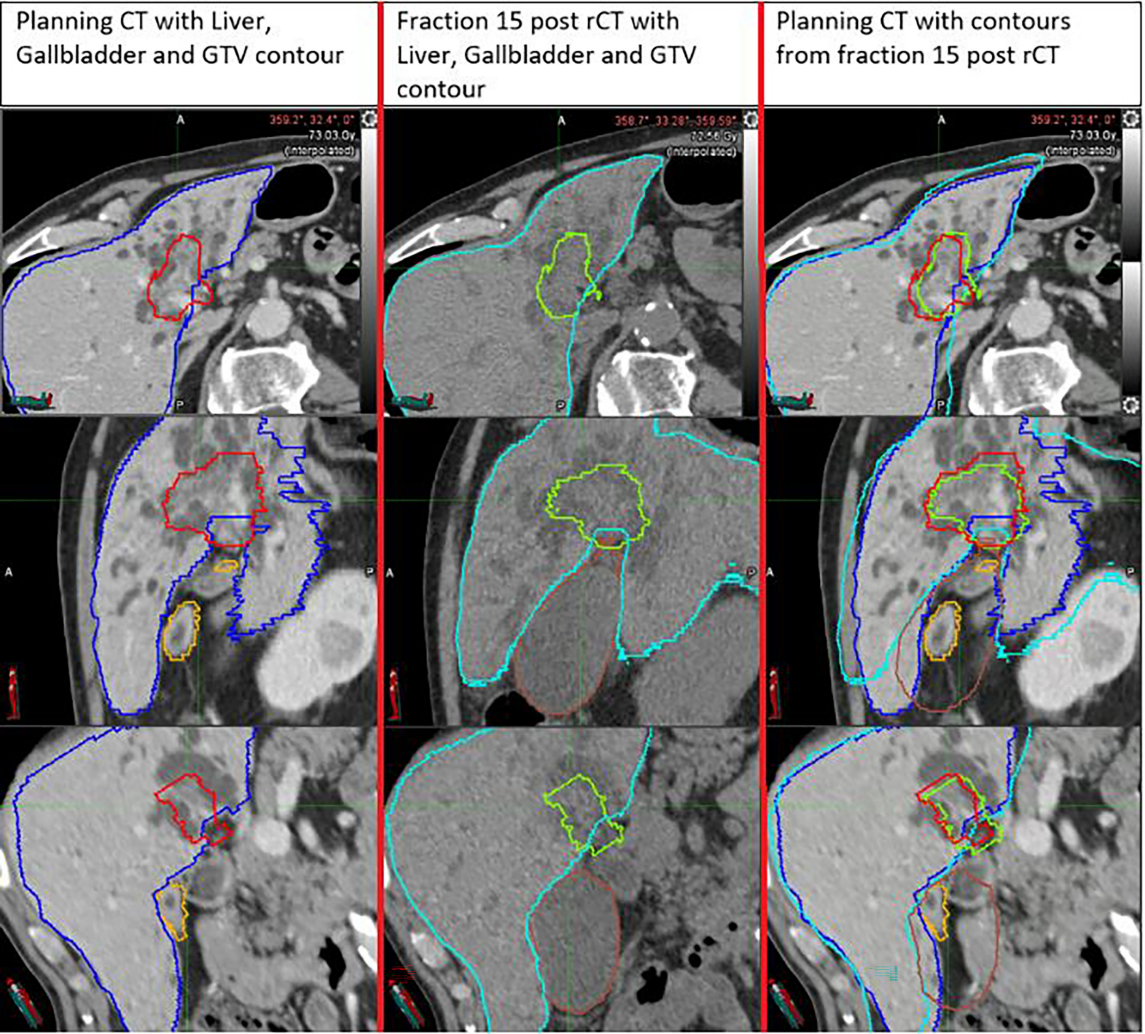
Example of a large PTV coverage loss (17.7%) in the post-rCT of the last fraction of patient 5, compared to pCT. The increased gallbladder volume pushed the fiducial away from the target, leading to less efficient tracking and considerable underdosage of the target.

Two of the greatest coverage losses were observed for patient 5 in the last treatment fraction (18.0% in pre-rCT and 17.7% in post-rCT). Inspection of the scans showed that these target losses were related to significant increases in the gallbladder volume during treatment (7.6 cc in the pCT vs. 61.3 in the pre-rCT and 79.8 cc in the post-rCT). Due to this effect, the fiducials were pushed further away from the target than in planning, causing a partial mismatch between the rCT PTV and the high dose volume delivered (**Figure 3**).

### 3.2 OAR dose deviations

**Figure 4:**
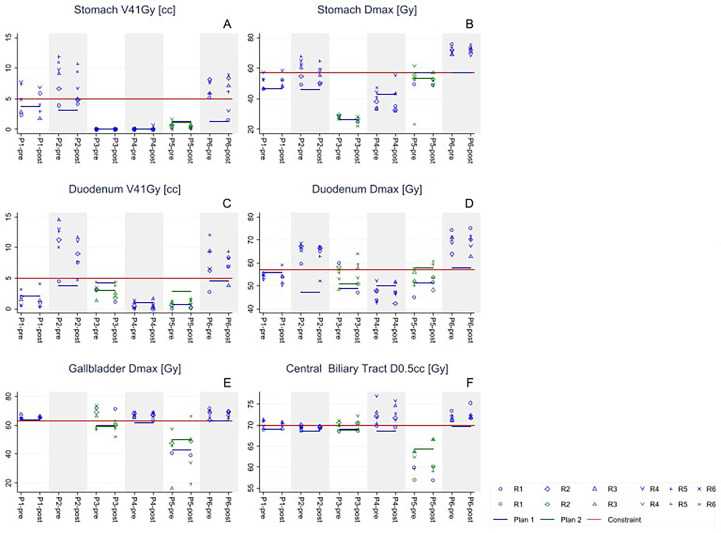
For all 6 study patients, P_i_, re-calculated stomach, duodenum, gallbladder and central biliary tract dose in repeat CT-scans, Rj, acquired pre- and post-dose delivery, compared to planned dose and planning constraints. For patients 3 and 5 there was an updated treatment plan during the fractionated treatment (Plan 2). Each panel analyzes a major constraint as described in the STRONG study (see **Table 2**).

**Table 2** shows an overview of the constraint violations observed. Although the 1st data column of **Table 2** suggests that there were some minor constraint violations for pCTs (< 1Gy), these violations were not present in the clinical plans generated and calculated with the Precision TPS. They were attributed to our use of the MIM software for data analysis, and were possibly related to small differences between MIM and Precision in DVH generation (M&M). Notwithstanding the relatively low planned target coverages (intended to protect OARs; see **Figure 1** and **Table 1**), pre-rCTs showed frequent and large OAR constraint violations for all OAR plan parameters (2nd data column of **Table 2** and **Figure 4**). For each of the main OAR plan parameters, 10 or more violations were recorded in the pre-rCTs; with a maximum of 21 violations out of 29 for the gallbladder Dmax (one patient did not have a gallbladder). On average, the six OAR plan parameters had constraint violations in 44.4% of pre-rCTs. There were considerable inter-patient differences.

**Table 2:** OAR constraint violations in planning and in repeat CTs in the patient population.

Structure parameter + constraint	pCT violations/N mean violation ± SD, range	pre-rCT violations/N mean violation ± SD, range	post-rCT violations/N mean violation ± SD, range
Stomach V41Gy ≤ 5 cc	0/6	13/35 3.0±2.0, [0.14,6.9]	9/35 2.8 ± 1.6, [0.9,5.7]
Stomach Dmax < 57 Gy	1/6 0.28	12/35 10.1± 5.9, [0.03,18.7]	10/35 10.3 ± 6.8, [0.7,18.7]
Duodenum V41Gy ≤ 5 cc	0/6	10/35 5.50 ± 2.71, [1.24,9.51]	10/35 3.7 ± 1.6, [1.8,6.6]
Duodenum Dmax < 57 Gy	1/6 0.9	15/35 9.0 ± 5.1, [0.5,17.3]	17/35 8.0 ± 5.0, [0.7,18.1]
Gallbladder Dmax ≤ 63 Gy	2/6 0.4 ± 0.3, [0.15,0.6]	21/29 4.5 ± 2.7, [0.7,10.3]	19/29 3.9 ± 2.1, [0.2,8.3]
Central biliary tract D0.5cc < 70 Gy	0/6	18/35 1.8 ± 1.6, [0.01,6.9]	17/35 2.1 ± 1.7, [0.3,5.7]

pCT, planning CT; pre- and post-rCT, repeat CT-scans acquired in daily fractions before and after dose delivery. Mean values and ranges only include scans that had a violation. N is the total number of observations.

Differences relative to planning in dosimetric OAR dose values were as large for pre-rCTs as they were for post-rCTs. Differences relative to planning were similar for pre- and post- rCTs (compare pre- and post-rCTs in **Figure 4**). This similarity is also visible in the last column of **Table 1**, which shows that the difference between pre- and post-rCTs was statistically significant only for stomach V41Gy.

## 4 Discussion

This is the first study to analyze inter- and intrafraction variations in doses delivered to targets and OARs in patients with pCCa. Patients were treated with robotic SBRT using implanted fiducials for respiratory tracking. Daily dose variations were assessed with an in-room diagnostic CT-scanner integrated into the treatment unit. Overall, planned target coverages were on average 11.9% below the intended 95%. Although, overall, the target doses in the rCTs deviated only slightly from the planned values, some PTV coverage losses were >10%. These differences in rCTs were related to displacements of the targets relative to the fiducials (non-rigid motion). Although the rCTs had many considerable OAR constraint violations, these were highly patient dependent. Differences between OAR pre- and post-rCTs were also patient dependent, and generally not statistically significant.

As differences between pre- and post-rCTs in OAR plan parameters were mostly not statistically significant while deviations between pre-rCTs and pCTs were frequently statistically significant and large, daily plan adaptation based on an acquired pre-rCT could be an interesting option to further explore. Daily plan adaptation has already been investigated for other abdominal tumors, such as those in the liver and pancreas (22, 23). It was shown in a recent study in which liver tumors were treated on an MR-Linac with and without daily replanning (24) that daily replanning led to a lower dose to the OARs and a higher target coverage. This procedure would need careful investigation, as it would increase the total treatment time, possibly resulting in (more) anatomical changes after scanning. This problem has already been highlighted for the MR-Linac, showing that the long intervals between scanning and treatment delivery may cause the position and filling status of healthy tissues to change, thereby reducing the target coverage and increasing the dose delivered to the OAR (25).

To our knowledge, no timing studies for daily re-segmentation and plan adaptation for pCCa have yet been performed, either for the Cyberknife or MR-linacs, or for the Ethos system for daily adaptive treatment, which was presented recently, and is based on cone beam CT scans (Varian Medical Systems, Inc) (26). Current MR-linac systems and the Ethos system are equipped with options for breath-hold treatment, but not for tracking. Compared to tracking (as available with the Cyberknife robotic treatment unit), this would enhance treatment time. The VMAT dose delivery of Ethos systems may help to deliver treatment faster than static beam treatments on MR-linacs. The Cyberknife uses a large number of non-coplanar beams to treat tumors, meaning that treatment times are relatively long. With regard to liver SBRT, convincing evidence has been found for enhanced dosimetric plan quality with non-coplanar configurations (27–29). Planning studies comparing coplanar with non-coplanar treatment for pCCa have not yet been published.

For treatment accuracy, the optimal system for external beam photon therapy for pCCa would probably be a unit that has both on-line MR-guidance and options for non-coplanar dose delivery and fast and high-quality daily plan adaptation. Such a system is currently not available. Due to favorable beam characteristics of proton beams, adequate dose distributions could possibly be generated with proton therapy, without a need for non-coplanar beams. For image-guidance, also here MR would be preferable, and developments on integration of MR are on-going (30). There is also work on going on fast and accurate plan adaptation in proton therapy, (31).

As an alternative to daily replanning, the Cyberknife system has an option named “dose shift,” which makes it possible to shift the full treatment plan if displacement of the target relative to the fiducials is detected; see **Figure 2** (19). While in principle this could avoid replanning, it might also result in undesired increases in OAR doses. To verify OAR doses, resulting dose distributions could be calculated in the acquired pre-rCT. Unfortunately, the dose shift on the Cyberknife cannot currently be used in combination with respiratory tracking, as needed for pCCa.

For tracking, this study used fiducial markers implanted close to the intrahepatic part of the tumor. Our analyses showed considerable target underdosages in rCTs, which were related to target displacements relative to these fiducials. While this may have been caused by non-rigid anatomy changes related to relatively large distances between fiducials and targets, it might also have been caused by inaccuracies in our rigid translation-rotation mapping of targets from pCTs onto rCTs (Materials and Methods section). To verify the quality of these mappings, we visually inspected the rCTs with mapped targets. In the five patients with a stent, there was both an excellent overlap between stents, and between soft tissues around the mapped GTV. These observations suggest that the observed large target underdosages were indeed caused by longer-range, non-rigid anatomy variations, and not by inaccuracies in the rigid mapping of targets with stents. With this excellent mapping of targets with stents, one option for further exploration – and thus a topic for further research – is tracking based on these stents rather than on implanted fiducials further away.

The only clinically relevant toxicity that was recorded in the STRONG trial was grade 3 cholangitis, which, despite the use of very strict planning constraints (32), was reported in five of the six patients (16). The only patient who did not experience this toxicity during the follow-up time of the study was patient 5, who had the lowest planned central biliary tract D0.5cc and even lower D0.5cc in most rCTs (**Figure 4F**). As a recent study has suggested, one possible explanation for the lack of toxicity in this patient is the absence of the stent itself (33).

Interestingly, although some patients had rather large and frequent constraint violations for duodenum, stomach, and gall bladder in rCTs, these did not result in dose-limiting toxicity (16) The constraints applied for planning were apparently strict enough for these patients to avoid toxicity. To confirm this absence of dose-limiting toxicity, larger clinical studies should be conducted.

This study had the following limitations. The first, which is related to the concept of the STRONG phase I clinical trial, is that only six patients were available for evaluation. However, as pre- and post- rCTs were acquired in six treatment fractions for each of these patients, a total of 70 evaluable scans were produced. Second, with regard to the dose deviations from planning in the rCTs, many of which were large, with very high percentages of constraint violations for OARs, we believe that our study clearly indicates the challenges involved in SBRT for pCCA, but also the options for further technical improvement. Third, although all repeat imaging in this study was performed with an in-room diagnostic CT-scanner, higher segmentation accuracy may have been achieved by in-room MR imaging. In-room CT-scans were acquired without IV contrast, which rendered target segmentation in rCTs unfeasible and OAR delineation more challenging. Fourth, as also described above, mapping of targets from pCTs to rCTs appeared to be accurate. Although tracking effectively compensated for intra-fraction respiratory motion, the lack of continuous imaging means that the intra-fraction dose variations unrelated to breathing should be regarded as lower limits. Fifth, only dosimetry in exhale was investigated. Finally, although the beam models used in the clinical dose engine and the standalone dose engine differed slightly, it was possible to resolve this difference with a minor correction of the calculated rCT doses.

## 5 Conclusions

During treatment for perihilar cholangiocarcinoma using robotic SBRT with respiratory tumor tracking, reductions in target coverage were greater than planned. These were attributed to non-rigid anatomy variations. Moreover, many of the daily OAR doses delivered were considerably higher than planned, indicating significant constraint violations. For most OAR plan parameters, intra-fraction changes were not statistically significant. The divergences between the doses delivered and those planned indicate opportunities for using advanced adaptive and motion-management approaches to enhance treatment quality.

## Data availability statement

The original contributions presented in the study are included in the article/**Supplementary Material**. Further inquiries can be directed to the corresponding author.

## Ethics statement

The studies involving human participants were reviewed and approved by Erasmus MC Medical Ethical Committee. The patients/participants provided their written informed consent to participate in this study.

## Author contributions

Conceptualization: AM and BH. Methodology: BH, CP, AM, MM, RB. Software: WD, MM. Validation: CP, WD, MM, YV, RB, BH, AM. Formal analysis: CP, WD, MM, YV, BH, AM. Investigation: CP, WD, MM, YV, BH, AM. Resources: AM, BH. Data curation: CP, WD. Writing—original draft preparation: CP. Writing—review and editing: CP, WD, MM, YV, RB, BH, AM. Visualization: CP, WD, YV, BH. Supervision: AM, BH. Project administration: AM. All authors have read and agreed to the published version of the manuscript. All authors contributed to the article and approved the submitted version.
